# The *vitellogenin* genes in *Cynops orientalis*: New insights on the evolution of the *vtg* gene family in amphibians

**DOI:** 10.1002/jez.b.23067

**Published:** 2021-06-25

**Authors:** Federica Carducci, Maria A. Biscotti, Adriana Canapa, Marco Barucca

**Affiliations:** ^1^ Dipartimento di Scienze della Vita e dell'Ambiente Università Politecnica delle Marche Ancona Italy

**Keywords:** amphibians, gene evolution, vertebrates, vitellogenin, phylogeny, transcriptomics

## Abstract

The vitellogenins (Vtgs) are glycolipophosphoproteins that play a key role in constituting nutritional reserves for embryo development in nonmammalian vertebrates. However, additional functional roles have been evidenced. These *vtg* genes are present in multiple copies, different in number and sequences in various vertebrate lineages. The comprehension of the *vtg* gene family evolutionary history remains a matter of intense interrogation for this field of research. In tetrapods, information about *vtg* genes are limited to few taxa. Up to date concerning amphibians, detailed studies have been conducted only in Anura. Therefore, in this study, to further increase knowledge about *vtg* genes in Amphibia class, the urodele *Cynops orientalis* (Amphibia: Caudata) was analyzed and four complete *vtg* sequences were obtained. Moreover, genomic data available for the caecilians *Microcaecilia unicolor* and *Rhinatrema bivittatum* (Amphibia: Gymnophiona) were also included. In these amphibians, our findings evidenced the presence of a *vtgI* sequence ortholog to that of tetrapods, absent in Anura. Moreover, microsyntenic, phylogenetic, and gene conversion analyses allowed postulating two hypotheses to explain the complex evolutionary history of this gene family.

## Research Highlights

- For the first time, the *vtgI* gene, absent in anurans, was characterized in caecilians and urodeles (Panel a).
- The different expression levels of *vtg* genes in female liver transcriptomes of *Cynops orientalis* suggested different functions (Panel b).

## Graphical abstract

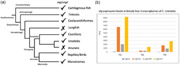

## 1 INTRODUCTION

The vitellogenins (Vtgs) are glycolipophosphoproteins constituted by a large multidomain apolipoprotein (Barucca et al., 2010; Canapa et al., 2007, 2012; Carducci et al., 2019; Verderame & Scudiero, 2017). The complete amino acid Vtg sequences have a signal polypeptide, a heavy chain lipovitellin including four subdomains (N‐sheet, α‐helix, C‐sheet, and A‐sheet), a phosvitin, a light chain lipovitellin, and a von Willebrand factor type D domain containing a βʹcomponent (βʹ‐c) and a C‐terminal coding region (Ct) (Carducci et al., 2019). These proteins play a key role in constituting nutritional reserves for embryo development in nonmammalian vertebrates. They are mainly synthesized in the female liver but also in males, at lower levels (Barucca et al., 2010; Canapa et al., 2007, 2012; Verderame & Scudiero, 2017). Their involvement in egg buoyancy, toxin transport and antimicrobial and antioxidant activities was reported in different species (Reading et al., 2017).

There is an increasing number of evidence about the presence of multiple *vtg* genes in different vertebrate lineages that makes difficult the reconstruction of *vtg* gene family evolution (Finn et al., 2009; Finn & Kristoffersen, 2007). In brief, as reviewed in our recent works (Biscotti et al., 2018; Carducci et al., 2019), up to three *vtg* genes have been found in nonteleost fishes. In particular, in lampreys (*Ichthyomyzon unicuspis* and *Petromyzon marinus*) a single gene has been reported while three *vtg* genes have been found in the elephant shark (*Callorhinchus milii*), in the spotted gar (*Lepisosteus oculatus*), and in the bichir (*Acipenser schrenckii*). In teleost fish, the *vtg* gene number is higher, with a maximum of eight genes in zebrafish (*Danio rerio*; Yilmaz et al., 2019). In basal sarcopterygians, three *vtg* genes have been identified in coelacanths (*Latimeria menadoensis* and *L. chalumnae*) (Canapa et al., 2012), and four genes in the lungfish (*Protopterus annectens*) (Biscotti et al., 2018). Three *vtg* genes have been described for birds and turtles, and a single gene has been retrieved in platypus (*Ornithorhynchus anatinus*) (Babin, 2008; Biscotti et al., 2018; Finn & Kristoffersen, 2007). Phylogenetic and microsyntenic analyses have revealed a clear orthology for *vtgI* genes of birds and turtles and *vtgC* genes of fish (Babin, 2008; Biscotti et al., 2018). For the remaining *vtg* genes tandemly organized in cluster, the orthology has not yet been defined (Carducci et al., 2019). In amphibians, past studies have considered only species belonging to the Anura order. In particular, three *vtg* genes have been described and their orthology to other tetrapod *vtg* genes is not clear (Brawand et al., 2008). Analyses performed on the *Xenopus* genome have not evidenced the presence of a *vtg* gene orthologous to *vtgI* of other tetrapods (Biscotti et al., 2018). No information is available about the other two orders: Caudata, the sister clade of anurans, and Gymnophiona, the early‐branching lineage of amphibians. In the present work, transcriptomic resources related to the urodele *C. orientalis* (common name: fire‐bellied newt) and genomic data of Gymnophiona were analyzed to get new insights onto the *vtg* evolutionary scenario in amphibians, a basal clade of tetrapods. Our results evidenced for the first time in amphibian species the presence of a *vtgI* gene showing orthology to those of birds and reptiles. Moreover, microsyntenic, phylogenetic, and gene conversion analyses allowed us to postulate two hypotheses explaining the evolutionary history of this intriguing gene family.

## 2 MATERIALS AND METHODS

Specimens of *C. orientalis* were obtained from a local dealer during the reproductive season. Three females and three males were anesthetized with MS222 at 2 g/l and sacrificed. All experimental procedures were approved by the Italian ethical committee Ministero della Salute (authorization n° 2E1BD.N.LYB) and all methods were performed in accordance with the relevant guidelines and regulations. Testes, ovaries, and female livers were dissected. Total RNA was extracted and used for sequencing using methodologies published by Biscotti et al. (2020).

### 2.1 *Cynops orientalis* sequences identification and characterization

Transcripts corresponding to *vtg* genes were retrieved from the high‐quality transcriptome assembly of *C. orientalis* published by Biscotti et al. (2020) using a tBLASTn sequence homology search. Two complete sequences and five partial transcripts were obtained. These latter were used to obtain two further *vtg* complete sequences. The missing portions were completed through a polymerase chain reaction (PCR)‐based approach starting from the complementary DNA synthesized from the female liver RNA samples processed in Biscotti et al. (2020). Reverse transcription was done using Superscript III First‐strand Reaction Mix (Thermo Fisher, Invitrogen) and random primers. PCRs were performed in a thermal cycler using Platinum Taq DNA Polymerase (Thermo Fisher, Invitrogen). The complete list of primers used in this study and the amplification profile are provided in Figure S1. PCR products were sequenced using Sanger sequencing technology. Pairwise distance between the obtained sequences was calculated using a tool in MEGA X (Kumar et al., 2018). The four nucleotide sequences were translated using EMBL/EBI Sequence Translation Tool (https://www.ebi.ac.uk/Tools/st/emboss_transeq/). Protein function was annotated through the Gene Ontology database using the web server PredictProtein (https://predictprotein.org/; Rost & Liu, 2003). These sequence data have been submitted to the GenBank databases under accession number MZ064525‐MZ064528.

### 2.2 Phylogenetic analyses

In the phylogenetic analysis, the four Vtg sequences obtained in this study for *C. orientalis* (Caudata order) and the five sequences collected from NCBI (https://www.ncbi.nlm.nih.gov/) for *Microcaecilia unicolor* and *Rhinatrema bivittatum*, two species belonging to Gymnophiona order, were used. In this analysis, additional eight amphibian sequences (four sequences of *Xenopus laevis* and three of *X. tropicalis* for Anura order and one sequence of *Andrias davidianus* for Caudata order) and 60 Vtg amino acid sequences belonging to other vertebrates were included. The complete list of accession numbers of sequences was reported in Table S1. The alignment was performed with ClustalOmega (https://www.ebi.ac.uk/Tools/msa/clustalo/) using default parameters. The phylogenetic analysis was carried out with MrBayes‐3.2 (Huelsenbeck et al., 2001). The Jones amino acid model (Jones et al., 1992) was identified by the MrBayes program (Ronquist et al., 2012) with a posterior probability of 1.00. 1,000,000 generations were run, and sampling conducted every 100 generations. Stationarity was defined as the condition where the standard deviation of split frequencies reached 0.003. The first 2500 trees were discarded as the burn‐in. The sequence of silver lamprey *I. unicuspis* was used as an outgroup. The adequacy of posterior samples taken from the Monte Carlo Markov Chain analysis was assessed estimating the effective sample size (ESS) using Tracer (Rambaut et al., 2018). ESS value was more than 200.

### 2.3 Gene conversion analysis

The program GENECONV (http://www.math.wustl.edu/sawyer/geneconv/gconvdoc.pdf; Sawyer, 1989) determines the extent of gene conversion in a set of sequences. It seeks protein segments for which a pair of sequences are sufficiently similar to suggest that gene conversion occurred. Inner fragments are evidence of a possible gene conversion event between ancestors of two sequences in the alignment. The output results are ranked by *p*‐values and presented in a spreadsheet manner. The alignment of Vtg amino acid sequences for each amphibian species was used as input and tested using GENECONV to look for gene conversion tracts. The analysis was conducted using default parameters exception made for/p (protein sequences);/w123 (internal random number generator);/lp (list of pairwise) setting 2,000,000 permutations; finally, as gscale/g0,/g2, and/g7 mismatch penalties were used (data not shown).

### 2.4 Microsyntenic analysis in basal sarcopterygians

The microsyntenic arrangement of *vtg* genes were obtained for the caecilian *M. unicolor* and the lungfish *Neoceratodus forsteri*. The strategy adopted consists in a BLAST analysis on the genome of interest using the “Whole Genome Sequencing Shotgun” tool. The identification of *vtg* genes and their upstream and downstream flanking genes allowed the comparison with coelacanth (basal sarcopterygian) and tetrapods available in Biscotti et al. (2018).

### 2.5 Gene expression analyses

*C. orientalis* transcriptomic trimmed reads were mapped against the four Vtg nucleotide sequences in CLC Genomics Workbench v.12 environment (Qiagen). The alignment between the *vtg* sequences and trimmed reads of the three female livers and the three female gonads was performed with the *RNA‐seq* mapping tool, setting mapping parameters as highly stringent (length fraction 0.9 and similarity fraction 0.9).

Gene expression levels were computed as transcripts per million (TPM) (Falcon & Gentleman, 2008), as this metric allows to efficiently compare gene expression levels both within and between samples. We followed the same strategy described in Biscotti et al. (2020), using a subset of 1694 unequivocal single‐copy orthologs.

## 3 RESULTS

Two complete *vtg* sequences and five partial transcripts were obtained from the high‐quality transcriptome of *C. orientalis*. Using a PCR‐based approach two additional *vtg* sequences were reconstructed from the partial fragments. The attribution to the *vtg* gene family was checked through a BLAST sequence similarity search. The four *vtg* transcripts were named vitellogenin *C. orientalis* I (*vtgCoI*), *vtgCoII*, *vtgCoIII*, and *vtgCoIV* and were of 5331, 5849, 5490, and 5479 bp long, respectively.

The pairwise distance was of about 46% between *vtgCoI* and the other three sequences and within these latter ranging from 27.6% to 29.4%. Moreover, *vtg* sequences were searched and identified in the sequenced genomes of two caecilian species available in public databases. A total of three *vtg* sequences for the *M. unicolor* and two for the *R. bivittatum* were retrieved and used for phylogenetic, gene conversion, and microsyntenic analyses.

**Figure 1:**
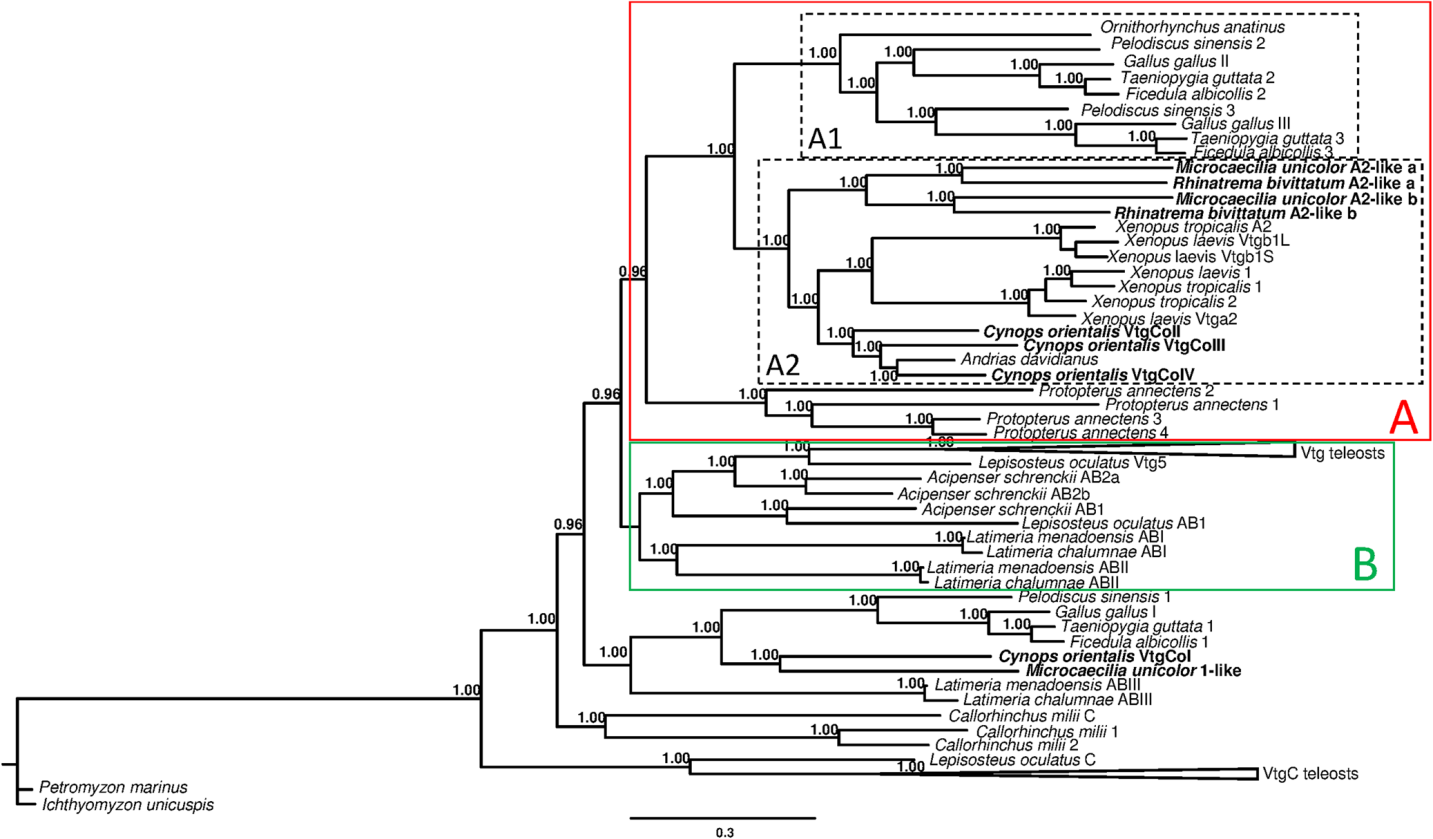
Phylogenetic analysis of vitellogenin amino acid sequences performed with Bayesian inference. The number beside nodes indicates posterior probability values (>0.95). Colored boxes marked with letters A (red) and B (green) indicate the two main clades described in the text. Moreover, the two subgroups identified in the Clade A are indicated using black dashed boxes and marked as A1 and A2, respectively. The sequence of silver lamprey *Ichthyomyzon unicuspis* was used as outgroup. Amphibian sequences analyzed for the first time are reported in bold [Color figure can be viewed at http://wileyonlinelibrary.com]

The phylogenetic analysis was based on a total of 77 amino acid sequences (24 species) whose 17 belong to 6 species of amphibians (Table S1). In the phylogenetic tree, two main clades can be observed: One characterized by the sequences of tetrapods together with those of lungfish *P. annectens* (Clade A) and the other constituted by sequences of *L. chalumnae* and *L. menadoensis*, *A. schrenckii*, *L. oculatus*, and teleosts (Clade B). Finally, in external position, three groups were present: VtgI sequences of tetrapods, Vtg sequences of *C. milii*, and VtgC sequences of teleosts. In particular, the VtgI sequences of tetrapods included that one found in the caecilian *M. unicolor* and the VtgCoI belonging to the salamander *C. orientalis* (Figure 1).

The relationships evidenced in the Clade A were of interest for this study. Indeed, one subgroup (A1) of this clade was composed of Vtg sequences of the turtle *Pelodiscus sinensis*, the birds *Gallus gallus*, *Taeniopygia guttata*, and *Ficedula albicollis*, and the mammal *O. anatinus*. The second one (A2) can be further divided into three subgroups corresponding to the Vtg sequences of Anura (*X. laevis* and *X. tropicalis*), Caudata (*C. orientalis* and *A. davidianus*) and Gymnophiona (*R. bivittatum* and *M. unicolor*) with these latter as sister group compared to the sequences of other amphibians. In particular, the Vtg sequences of Anura, Caudata, and Gymnophiona and those of birds did not group following the species phylogenetic relationships but the sequence orthology.

Analyses using GENECONV were conducted to search possible gene conversion events that occurred in amphibians (*C. orientalis*, *M. unicolor*, *R. bivittatum*, *X. tropicalis*, *X. laevis*). Setting the gscale (mismatch penalty) parameter as 2, for *C. orientalis* results evidenced one region of 158 residues length between VtgCoIII and VtgCoIV and a region of 327 residues in VtgCoII that might have undergone to gene conversion. For *X. tropicalis*, a fragment of 89 residues length was evidenced between Vtg1 and Vtg2. For *X. laevis*, *M. unicolor*, and *R. bivittatum* relevant tracts in which gene conversion events might have occurred were not evidenced. Setting the mismatch penalties as 0 and 7 no further tracts that underwent gene conversion were evidenced, exception made for *X. laevis* in which two regions of 40 and 48 residues were detected between Vtga2 and Vtg1. All results here reported had *p*‐values less than 0.05. Results involving regions having small lengths were not taken into account.

**Figure 2:**
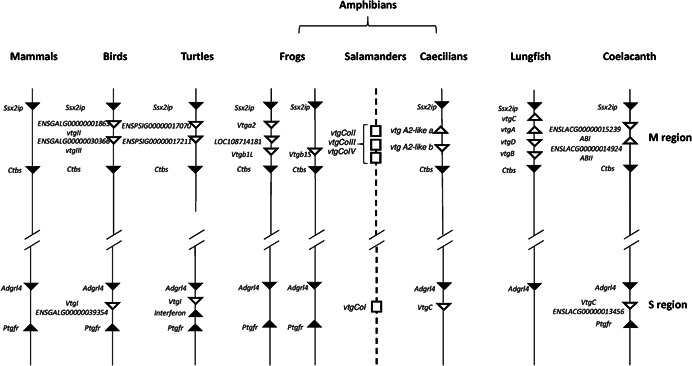
Microsyntenic arrangements of *vitellogenin* genes in tetrapods and basal sarcopterygians (lungfish and coelacanth). Triangles indicate genes and their direction. White filled triangles are *vtg* genes and black‐filled triangles are related to upstream and downstream flanking genes. Note that gene distances are not in scale. For salamanders (*Cynops orientalis* genome not yet sequenced) the dashed line and white rectangles indicate the position of *vtg* genes hypothesized on the basis of phylogenetic results

Previous studies have evidenced that the *vtg* genes are located in two regions (Babin, 2008), named M (multiple *vtg* genes) and S (single *vtg* gene) (Biscotti et al., 2018). In this study, the arrangement of *vtg* genes was investigated in the genome assemblies of lungfish and caecilians (Figure 2) and compared with coelacanth and tetrapods available in Biscotti et al. (2018). The microsyntenic analysis revealed that the three *vtg* genes identified in caecilians are located two in the M region and one in the S region. Regarding the lungfish *N. forsteri* the four *vtg* genes were located in the M region. The presence of a *vtg* gene in the S region in lungfish cannot be excluded since our search on available genome data did not allow to identify the flanking genes suggesting that this region is not correctly assembled. In Figure 2 the microsyntenic arrangement of *C. orientalis*, whose genome is not available, was hypothesized on the basis of the results obtained in the phylogenetic analysis.

**Figure 3:**
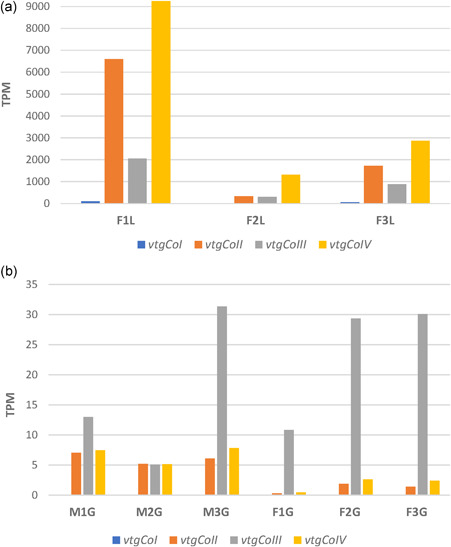
Gene transcriptional levels of *Cynops orientalis vitellogenin* genes. The graph A shows the transcriptional activity of the four *vtg* genes in three female livers; the graph B shows the transcriptional activity in male and female gonadal tissues. Values are reported as transcripts per kilobase million (TPM). F1L, F2L, and F3L are referred to female livers. M1G, M2G, and M3G are referred to male gonads and F1G, F2G, and F3G are female gonads [Color figure can be viewed at http://wileyonlinelibrary.com]

The transcriptional activity of four *vtg* genes identified in *Cynops* was evaluated through RNA‐seq. The analysis performed on data obtained from three female livers showed a remarkable activity of *vtgCoII*, *vtgCoIII*, and *vtgCoIV* (Figure 3a). In particular, in all samples, the *vtgCoIV* TPM values were the highest, followed by those of *vtgCoII* and *vtgCoIII*. Overall, in liver tissues, the *vtgCoI* was characterized by negligible expression values compared to other *vtgs*. Different expression levels observable in the three female livers can be explained following histological observations made for these female gonads and published in Biscotti et al. (2020) in which clear asynchronous developmental stages among samples emerged.

Transcriptional activity of *vtgs* was found also at gonadal level, although with lower TPM values, both in the case of male and female individuals (Figure 3b). In gonads, the expression of *vtgs* is mainly referred to *vtgCoIII*. Overall, it can be affirmed that the *vtgCoI* was not expressed at gonadal level. The Gene Ontology functional annotation of isolated Vtg sequences evidenced a nutrient reservoir activity for the VtgCoII and VtgCoIV and a lipid transfer activity for the VtgCoIII. No functional annotation was obtained for VtgCoI (data not shown).

## 4 DISCUSSION

The analysis of *C. orientalis* transcriptome coupled with sequence reconstruction through PCR approach allowed to identify four sequences attributable to the *vtg* gene family. The phylogenetic analysis clearly showed the orthology of one sequence to the VtgI of tetrapods together with one sequence identified in the caecilian *M. unicolor*. The presence of this gene in two orders of Amphibia demonstrated that this gene was already present in the common ancestor of this clade and its absence in anurans was due to secondary loss. These findings were confirmed at microsyntenic level in caecilians whose genome was available.

In previous works (Babin, 2008; Biscotti et al., 2018), the microsyntenic arrangement of *vtg* genes revealed that in sarcopterygians these genes are located into two regions, named M and S region. While a single gene (*vtgI*) is located in the S region, multiple genes are present in the M region. The microsyntenic analysis conducted in the present work on caecilian *M. unicolor* showed the same distribution of *vtg* genes also in this order of amphibians. As regard *vtg* genes present in the M region their orthology is still unclear. In 2008, Babin has hypothesized the orthology of two genes present in M region in the major part of tetrapods. Brawand et al. (2008) and Biscotti et al. (2018) have explained the current distribution of *vtg* genes located in this region as the result of independent lineage‐specific duplications from a unique ancestral gene on the basis also of phylogenetic analyses. Indeed, while in the phylogenetic tree the sequences of VtgII of birds and those of the turtle *P. sinensis* constituted a paraphyletic group to that of the VtgIII of the same species evidencing a clear orthology, the sequences of amphibians clusterized for order underlining the missing of orthology between the Vtgs of amphibians and other tetrapods.

**Figure 4:**
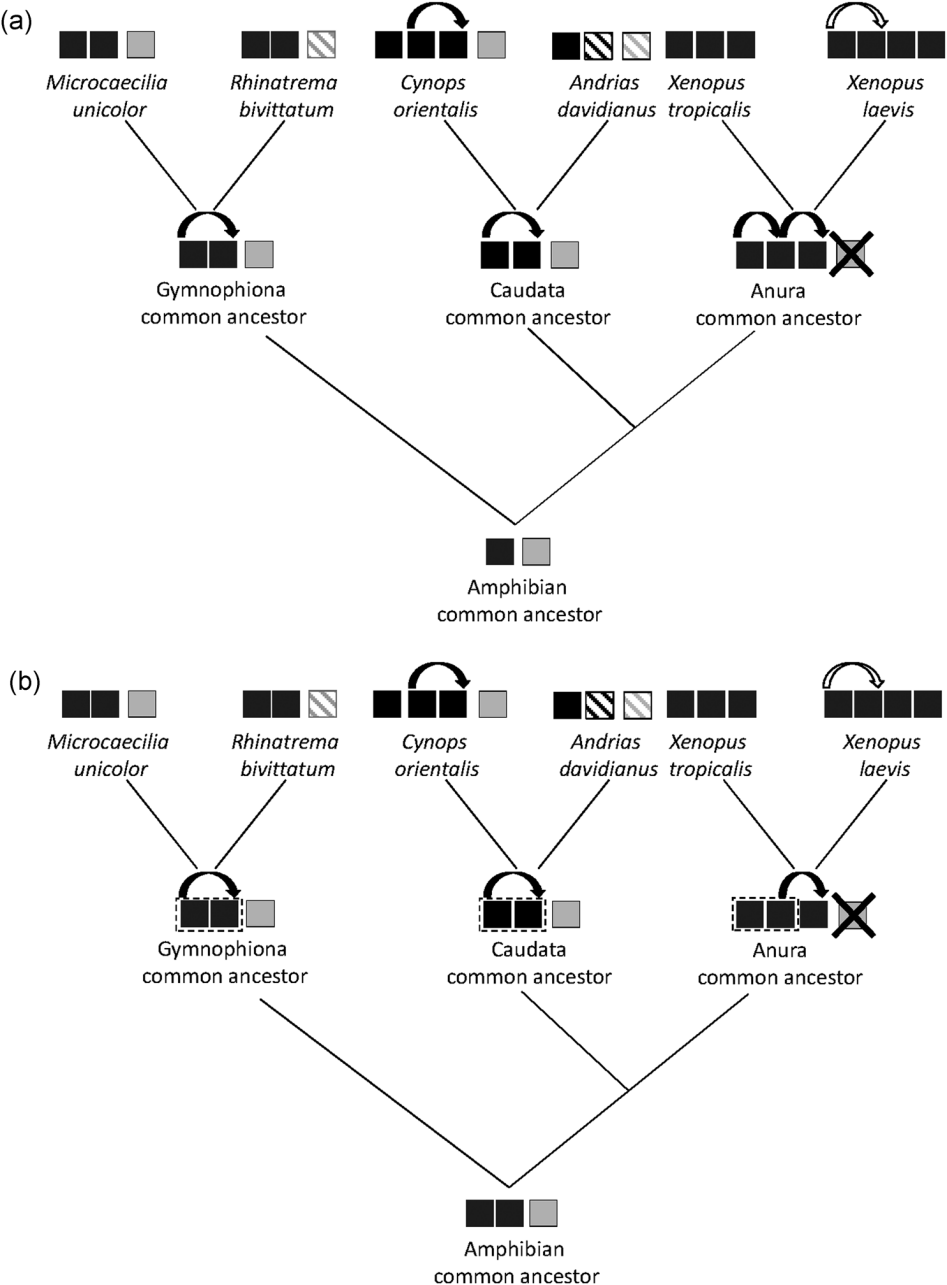
Schematic representation of the two hypotheses proposed for the evolution of *vitellogenin* gene family in amphibians. In the upper scheme (a) the evolutionary hypothesis based on the results obtained from phylogenetic analysis is showed; in the lower scheme (b) evolutionary hypothesis, in which gene conversion events have affected the phylogenetic relationship between sequences, is illustrated. Their detailed explanation is provided is the text. Black‐filled squared are vitellogenin genes located in the M region and gray‐ filled square represents the single gene located in the S region (*vtgI*). Black and gray striped squares are referred to hypothesized *vtg* genes whose presence cannot be excluded. Crossed square indicates the loss of the *vtg* gene in locus S. Black curved arrows above filled squares indicate duplication events. White curved arrow indicates polyploidization‐related duplication event occurred in *Xenopus laevis*. Dashed rectangles indicate hypothesized gene conversion events

However, it has been hypothesized that the nonallelic gene conversion (NAGC) phenomena could explain the failure of phylogenies to reconstruct the duplication of the *vtg* genes located in the locus M occurred in the amphibian ancestor (Braasch & Salzburger, 2009). NAGC is the copying of a genetic sequence from a “donor” region to an “acceptor” in which the donor and the acceptor are at distinct genetic loci. These recombination events can easily occur when the paralogous sequences are erroneously aligned because of their high similarity. This is common in recent tandem gene duplicates while, over the time, mutations accumulate and sequences diverge leading to the disappearance of this phenomenon (Harpak et al., 2017). The occurring of NAGC between genes tandemly arranged can determine their clusterization due to the homogenization of the sequences rather than clusterization in phylogenetic analysis with ortholog sequences present in other species (Braasch & Salzburger, 2009). Therefore, regarding the *vtgs* of amphibians, two hypotheses can be suggested (Figure 4). According to the first hypothesis, in the common ancestor of amphibians two *vtg* genes were present: *vtgI* and *vtgII/III* ancestral genes. The *vtgI* gene is currently present in the Gymnophiona and Caudata lineages, while this gene was lost in anurans. The vtgII/III was undergone to a tandem duplication independently in all three amphibian orders. Therefore, caecilians show three genes and the newt *C. orientalis* has four genes, the *vtgI* and three genes derived from additional duplications of the *vtgII/III* gene. Data available do not allow to know if this condition is shared in urodeles. In anurans, the presence of three genes is due to two duplication events from the *vtgII/III* gene in the ancestor of this clade. In *X. laevis*, a fourth gene is derived by a polyploidization event occurred in the genome of this species. This model is in agreement with the phylogenetic tree and excludes that NAGC events have influenced phylogenetic relationships between sequences (Figure 3a). In the second hypothesis, the amphibian ancestor had three genes: *vtgI*, *vtgII*, and *vtgIII*, the latter two derived from a duplication of an ancestral gene. Subsequently, when the *vtgII* was highly similar to the *vtgIII*, independent gene conversion events occurred in the ancestors of the three amphibian orders. Therefore, in amphibians, three genes are expected as currently observed in Gymnophiona. In *C. orientalis* and probably in all urodeles a further duplication led a fourth gene. A similar event occurred in anurans in which, however, the *vtgI* was lost. Past gene conversion events occurred in the ancestor are not efficiently traced due to the increase of sequence divergence. However, this second hypothesis is supported by the analysis performed with GENECONV that evidenced possible gene conversion events between the *vtg* genes. The presence of three genes also in reptiles and birds allowed us to hypothesize that this condition was already present in the ancestor of amniotes. Therefore, the duplication event that led to *vtgII* and *vtgIII* might be dated at 400 Mya (or even before) in the common ancestor of tetrapods.

Recently the presence of multiple *vtg* genes has been correlated with several functions, beside as source of yolk nutrients for early developmental stages (Carducci et al., 2019). For example, in acanthomorph fish, the heavy chain of VtgAa lipovitellin is highly degraded during oocyte maturation, producing a pool of free amino acids that has an effect on oocyte hydration and egg buoyancy (Finn & Kristoffersen, 2007). In *Takifugu pardalis*, a Vtg subdomain is able to bind and transfer tetraodotoxin from liver to ovary where is accumulated in eggs as a repellent against predators and as pheromone able to attract males (Yin et al., 2017). Furthermore, other papers evidenced also antimicrobial activity (Liu et al., 2009; Shi et al., 2006; Zhang et al., 2005) and antioxidant activity for Vtg (Li & Zhang, 2017; Sun & Zhang, 2015).

The different expression levels of *vtg* genes in *Cynops* suggested that they might be involved in different functions. Indeed, the *vtgCoII* and *vtgCoIV* showed a high expression in livers of sexual mature females suggesting a role in yolk formation as also confirmed by functional annotation. The *vtgCoIII* showed a lower expression in the liver compared to *vtgCoII* and *vtgCoIV* and its activity was also detected in male and female gonads. Overall, the expression of *vtgs* in gonads was due to authosynthesis, a process that occurs in gonads contrarily from heterosynthesis that occurs in the liver. The not gender‐related expression of *vtgs* has been proposed to not be correlated to nutritional functions (Carducci et al., 2019). The *vtgCoIII*, originated with *vtgCoIV* from a duplication event, as also evidenced in the phylogenetic tree in which they group together, might have acquired a new function in the gonads of both sexes. Indeed, the annotation with Gene Ontology showed a “lipid transfer activity” for this sequence. In the analyzed tissues, no transcriptional activity was evidenced for *vtgCoI* suggesting that its role might be played in a different developmental stage or in a different tissue.

## 5 CONCLUSIONS

Solving the puzzle of the evolutionary history of *vtg* gene family remains a challenge for this study field. Certainly, past studies conducted on basal sarcopterygians provided precious knowledge to comprehend the number and genomic organization of these genes in the common ancestor of tetrapods. However, in this taxon information about *vtg* genes are limited to few species with lineages not yet examined. Therefore, analyses here conducted, taking into account species belonging to urodeles and caecilians, increased our knowledge in the Amphibia class. Our results evidenced that the *vtg* genes number varies between evolutionary lineages of this clade. Moreover, the identification of homologous genes to *vtgI* of tetrapods in Gymnophiona and Caudata led us to affirm that this gene was already present in the common ancestor of this class and that Anura experienced a secondary loss. Expanding the data set to other tetrapod species will certainly contribute to define dynamics underlying the evolution of this gene family and functional studies will be necessary to definitely comprehend the roles of the duplicated *vtg* genes.

## CONFLICT OF INTERESTS

The authors declare that there are no conflict of interests.

### PEER REVIEW

The peer review history for this article is available at https://publons.com/publon/10.1002/jez.b.23067

## Supporting information

Supporting information.

Supporting information.

## Data Availability

All sequences included in the study are available in the public data bank and accession numbers are reported in Table S1.
