# Supplementary figures and images for: The vitellogenin genes in Cynops orientalis: New insights on the evolution of the vtg gene family in amphibians
Source: J Exp Zool B Mol Dev Evol. 2021 Jun 25;336(7):554–61. doi: 10.1002/jez.b.23067 (PMC8596760; doi:10.1002/jez.b.23067)

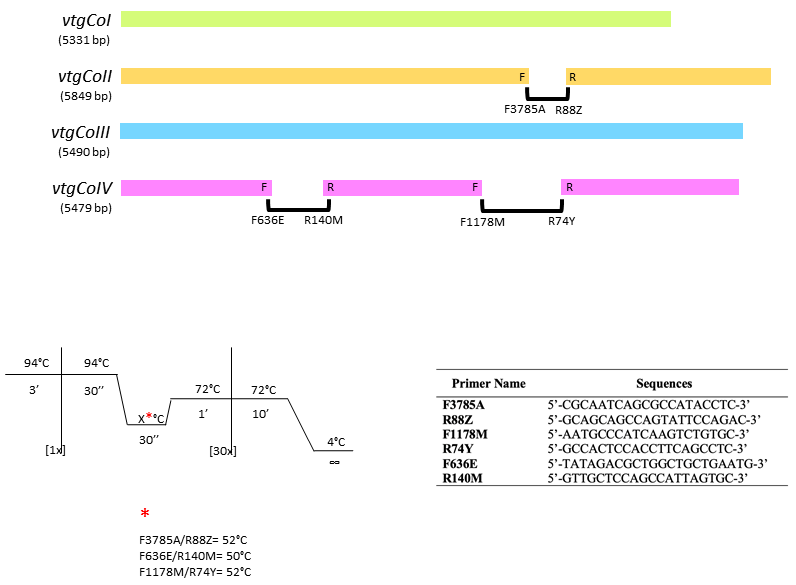

Supplement: Supplementary file 1 — Supporting information. [file JEZ-336-554-s002.tif]
